# Retinoic acid differently modulates NOD1/NOD2-mediated inflammatory responses in human macrophage subsets

**DOI:** 10.3389/fimmu.2025.1609763

**Published:** 2025-07-01

**Authors:** Hala Ahmad, Ahmad Alatshan, Eduárd Bíró, Szilvia Benkő

**Affiliations:** ^1^ Laboratory of Inflammation-Physiology, Department of Physiology, Faculty of Medicine, University of Debrecen, Debrecen, Hungary; ^2^ Doctoral School of Molecular Cell and Immune Biology, Faculty of Medicine, University of Debrecen, Debrecen, Hungary

**Keywords:** retinoic acid, NOD1, NOD2, NOD-like receptor, vitamin A, inflammation, macrophage, cytokine

## Abstract

Macrophages are indispensable in homeostasis and innate immune responses in multiple tissues, while their polarization and functional characteristics are determined by the activating stimuli and their tissue microenvironment. The vitamin A derivative retinoic acid shows inhomogeneous distribution among the tissues and has an important modulatory role in inflammatory responses. However, its effects on the cytokine secretion induced by the cytosolic pattern-recognition receptors NOD1 and NOD2 are unclear. In our study, we used human monocyte-derived macrophages differentiated in the presence of GM-CSF or M-CSF to generate inflammation inducing (GM-MФ) or inflammation resolving (M-MФ) cells, respectively. We activated the cells with either a NOD1- or NOD2 specific agonist and, using ELISA, we determined the pattern and dynamics of cytokines secreted by the macrophage subpopulations. Furthermore, we studied the effect of all-trans retinoic acid (ATRA) pre-treatment on the NOD1- and NOD2-induced cytokine release. Our comparative analysis shows subpopulation-characteristic pattern of cytokine secretion, as GM-MФ produce significantly higher pro-inflammatory IL-6, IL-8, TNF-α and IL-1β, while M-MФ secret higher anti-inflammatory IL-10. However, IL-18 and IFNβ secretion was comparable between the MФ subpopulations. We also show for the first time that ATRA has marked impact on cytokine secretion triggered by NOD1 and NOD2. Importantly however, the ATRA-induced changes of cytokine secretion follow opposite tendency in two MФ subpopulations. In conclusion, these results show that NOD1/NOD2-induced cytokine secretion by macrophage subsets is highly context-dependent and our results highlight the importance of the retinoic acid content of the local tissue environment in shaping macrophage function in health and disease.

## Introduction

Retinoic acid (RA) is the most prevalent and metabolically active vitamin A derivative in the human body that regulates a wide range of biological processes including cell proliferation and differentiation, embryogenesis and development, furthermore it supports the homeostasis of various tissues (1). The pleiotropic effect of RA are mediated by nuclear receptors either via genomic mechanisms by regulating transcription processes; or via non-genomic mechanisms by directly modifying the activity of various signal transduction pathways (2). Importantly, RA has also been shown to play a crucial role in the modulation of various immune functions. These include the differentiation and maturation of immune cells of both the innate- and adaptive immune system, such as lymphocytes, dendritic cells and macrophages (3, 4). Specifically in macrophages, RA has been implicated in various functions including phagocytosis, efferocytosis as well as balancing inflammatory responses through shaping cytokine production (3, 5, 6). Importantly, the modulatory effect of RA on inflammatory responses is highly context dependent as it is affected by the cell type, the stimuli and the tissue microenvironment.

Macrophages (MФs) are indispensable effector cells to modulate inflammatory responses. They form a diverse population of innate immune cells that are present in various tissues and organs where they either provide homeostatic functions as resident MФs, or support protective mechanism as infiltrating MФs by inducing or resolving inflammatory responses (7). Importantly, MФ inflammatory activity involves a complex molecular network including the cooperative crosstalk of pattern recognition receptors, signaling cascades and cytokine release. It is widely recognized that NOD-like receptors (NLRs), a family of cytosolic pattern recognition receptors that regulate various innate immune response, are highly involved in MФs functions (8).

NOD1 and NOD2 are prominent members of the NLR family as they were the earliest to be identified and characterized among the mammalian NLRs, but their roles in the inflammatory responses of MФ subsets are unknown. NOD1 and NOD2 were initially described to sense conserved, but distinct motifs of bacterial peptidoglycan (PGN), however, they can be activated by non-bacterial microorganisms (such as viruses, parasites and fungi) as well as danger-associated molecules (such as sphingosine-1-phosphate released during ER stress (9). Following activation, NOD1 and NOD2 participate in the regulation of cellular processes including metabolism, autophagy, cell death (10, 11) and the secretion of various cytokines (12–14).

Accumulating evidence shows that cytokine secretion by activated myeloid cells is highly modulated by RA (1). Accordingly, ATRA has been reported to enhance anti-inflammatory IL-10 secretion, but suppress IL-12 and TNF-α secretion in LPS-activated THP-1 cell line and splenic MФs (15, 16); while in human alveolar MФs and THP-1 cell line, LPS-induced IL-β expression is enhanced by ATRA (17, 18). Furthermore, we reported that in M-MФs, the LPS–induced IL-6 and IL-1β secretion is enhanced, while IL-10 is down-regulated by ATRA (19). While both NOD1 and NOD2 are important pattern recognition receptors that ignite inflammatory responses, including cytokine secretion in MФs, and the local microenvironment of MФs in several tissues may contain substantial amount of RA, the effect of RA on NOD1- and NOD2-mediated inflammatory cytokine and chemokine release has not been addressed before. Thus, we hypothesized that ATRA may modulate MФ inflammatory responses by interfering with NOD1/NOD2-induced cytokine responses in different MФ subsets.

To this end, we carried out a comparative analysis of typical inflammatory cytokine secretion induced by NOD1 and NOD2 in two distinct subpopulations of human monocyte-derived MФs that are differentiated in the presence of either GM-CSF or M-CSF, and that are commonly used *in vitro* models of pro-inflammatory- (GM-MФs) or pro-resolving cells (M- MФs), respectively. We focused on a few cytokines that are well-established to mediate the functional responses of different MФ populations as typical pro-inflammatory cytokines (IL-1β/IL-18, IL-6, TNF-α), or anti-inflammatory and pro-resolving cytokines (IL-10, IFNβ) (20, 21). IL-8 was specifically selected as a well-known downstream target of NOD1/NOD2 activation (22, 23). We show that the activation of NOD1 or NOD2 results in the release of different cytokine patterns by the two MФ subpopulations. Furthermore, we report for the first time that ATRA highly modulates cytokine secretion triggered by NOD1 and NOD2. Importantly however, the ATRA-induced changes of cytokine secretion by the two MФ subpopulations follow opposite tendency.

## Materials and methods

### Ethical statement

Buffy coat from healthy blood donors was provided by the regional National Blood Transfusion Service. The procedure was documentary approved by the Director of the National Blood Transfusion Service (Permit number: OVSZK/1678-2/2024/3090). The study and all experimental protocols were in accordance with the Regional and Institutional Ethics Committee of the University of Debrecen (Debrecen, Hungary). and the Regional and Institutional Ethics Committee of the University of Debrecen, Hungary.

### Monocyte isolation

Peripheral human blood mononuclear cells (PBMCs) were extracted from buffy coats using density gradient centrifugation with Ficoll Paque PLUS (GE Healthcare Life Sciences, Little Chalfont, United Kingdom). Then, monocytes were isolated by positive immunomagnetic cell selection using CD14 microbeads according to the manufacturer’s instruction (Miltenyi Biotec, Bergisch Gladbach, Germany).

### Macrophage differentiation

The isolated monocytes were cultured in RPMI 1640 medium (Sigma-Aldrich, St. Louis, MO, USA) supplemented with 2 mM L-glutamine, 10% heat-inactivated FBS, 100 U/mL penicillin-streptomycin and differentiated with either M-CSF (50ng/ml) or GM-CSF (80ng/ml) (PeproTech, Rocky Hill, NJ, USA). Cells were seeded in 24-well plates at a concentration of 1.1 x 10^6^ cells/mL at 37°C and 5% CO2. Half of the culture media was replaced with fresh media containing M-CSF or GM-CSF on day 2. The cells were differentiated for 5 days.

### Macrophage treatment

Macrophages were treated on Day 5 with C14-Tri-LAN-Gly (NOD1 specific agonist) (500ng/ml) (InvivoGen, San Diego, CA, USA) or L18-MDP (NOD2 specific agonist) (100ng/ml) (InvivoGen, San Diego, CA, USA) for the indicated time points. Where indicated, cells were pretreated with ATRA (1uM) for 4 hours (Sigma-Aldrich, St. Louis, MO, USA).

### Cytokine secretion measurements

Cytokine secretion levels were quantified using enzyme-linked immunosorbent assay (ELISA) according to the manufacturer’s instructions, IL-1β, IL-8 and TNF-α were measured using ELISA kits from BD Biosciences (San Diego, CA, USA). IL-18, IL-6, IL-10 and IFNβ were measured using ELISA kits from R&D Systems (Minneapolis, MN, USA). A microplate reader was used at 450 nm absorbance to determine cytokine levels (FlexStation 3, Molecular Devices, Sunnyvale, CA, USA). The minimum detectable levels were 3.9 pg/mL for IL-1β, 3.1 pg/mL for IL-8, 7.8 pg/mL for TNF-α, 11.7 pg/ml for IL-18, 9.38 pg/mL for IL-6, 31.3 pg/mL for IL-10 and 7.81 pg/mL for IFNβ.

### Statistical analysis

Results are presented as mean ± standard deviation (SD). Statistical analysis was conducted using One-way ANOVA followed by Tukey’s *post hoc* comparison. GraphPad Prism (version 8.4) was used for data analysis (GraphPad Software, San Diego, California, USA). Differences are considered significant at p value less than 0.05. In the figures, the asterisk (*) sign indicates significant difference between the NOD1/NOD2-treated and the control group at one given time-point. The hash symbol (#) indicates statistically significant difference between M- and GM-MФs at a given time-point.

## Results

### NOD1 differently activates human MФ subpopulations

**Figure 1:**
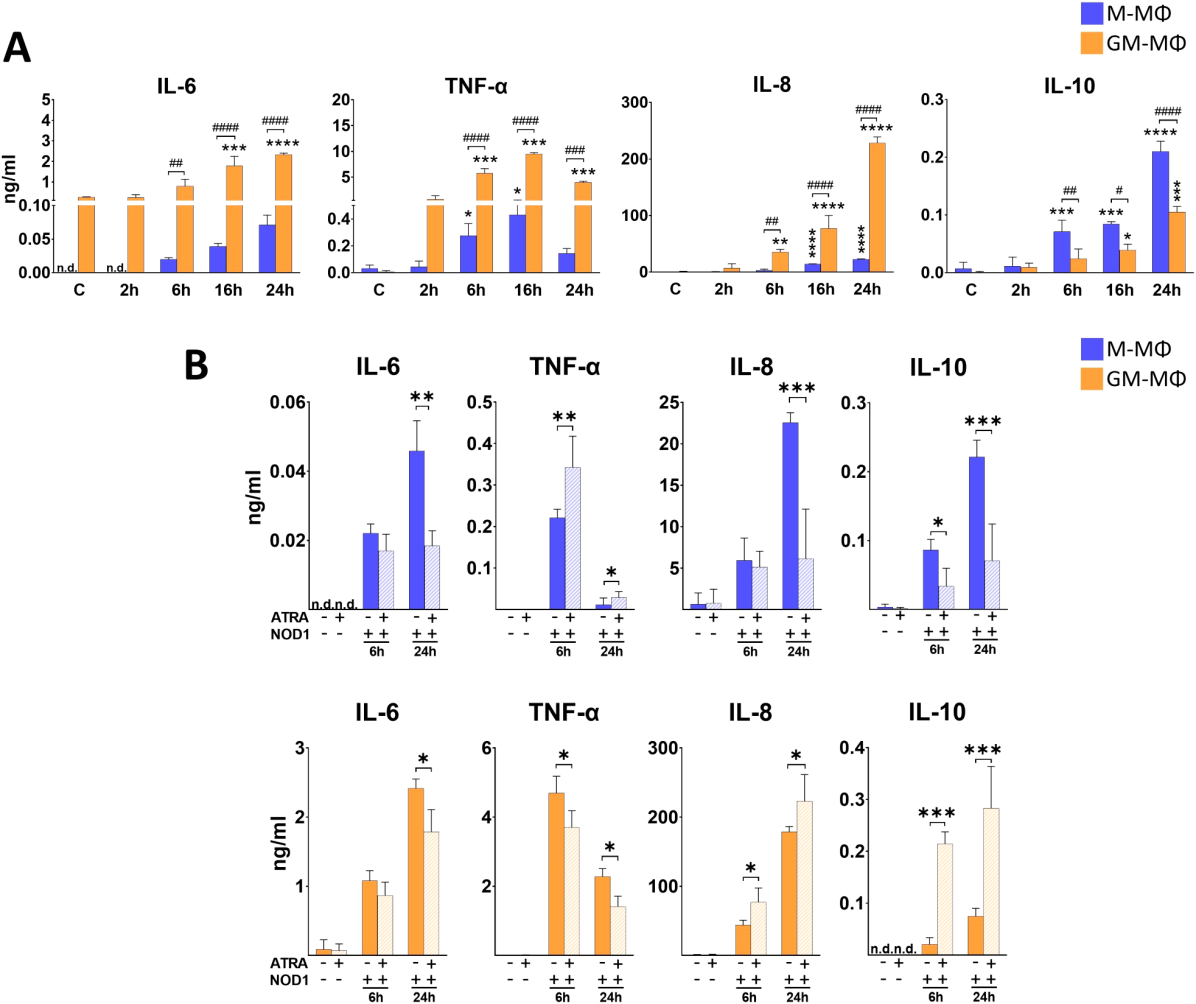
Secretion of IL-6, TNF-α, IL-8 and IL-10 following NOD1 activation. M-MФ and GM-MФ were treated with C14-Tri-LAN-Gly (NOD1 agonist, 500 ng/ml) for the indicated time points. Control cells were treated with the same amount of vehicle as the activated cells. Cytokine secretion was measured from the supernatant using ELISA. **(A)** Time-kinetics of cytokine secretion. **(B)** Cells were pretreated with ATRA (1µM) for 4 hours and then stimulated with C14-Tri-LAN-Gly for 6 and 24 hours. Results were obtained from at least four healthy donors. All results are shown as means ± SD. (^*^p < 0.05, ^**^p < 0.01, ^***^p < 0.001, ^****^p < 0.0001, ^#^p < 0.05, ^##^p < 0.01, ^###^p < 0.001, ^####^p < 0.0001; n.d., not detected).

Activation of NOD1 triggers the induction of various signaling pathways via RIPK2, and results in secretion of different inflammatory cytokines (24). To get a better insight into the dynamics of NOD1-induced cytokine secretion by the human monocyte-derived MФ subpopulations, cells were treated with NOD1 specific agonist (C14-Tri-LAN-Gly), and supernatant was collected at different time-points for ELISA measurements. The comparative analysis between the MФ subpopulations revealed significant differences in cytokine secretion (**Figure 1A**). While we measured a dynamic increase in IL-6 for both MФ types during the 24 h time interval, in TNF-α secretion we observed a peak at 16 h in both subpopulations. Nevertheless, the secretion of both cytokines was significantly higher in the supernatants of GM-MФs compared to M-MФs. We observed similar tendency in IL-8 chemokine secretion, as we measured gradually increasing, high level of IL-8 production by GM-MФs following NOD1 agonist treatment, while it was significantly less in the supernatant of M-MФs. Importantly, the opposite tendency was observed for the anti-inflammatory IL-10. While IL-10 was induced in both MФ subpopulations, IL-10 release was significantly stronger in M-MФs compared to GM-MФs. Though weakly expressed, but similar tendency was observed for IFNβ, an upstream regulatory cytokine of IL-10 (**Supplementary Figure 1A**). These results indicate that while NOD1 induces cytokine secretion, it results in a different cytokine profile by the different MФ subsets.

### NOD1-induced cytokine secretion is differently modified by ATRA in the MФ subpopulations

To determine whether ATRA has any modulatory effect on NOD1-induced cytokine secretion, cells were pretreated with ATRA, and supernatant was collected at various time-points following NOD1 activation. Of note, though we detected the same tendency of ATRA effect on cytokine secretion at each time-points (2h, 6h, 16h, 24h), we decided to only present an early (6h) and a late (24h) time point in the manuscript (2h and 16h data are not shown) (**Figure 1B**). Compared to the non-treated controls, ATRA treatment alone did not change the secretion of cytokines at any time-points. In the presence of ATRA, we found significantly decreased secretion of IL-6 in both NOD1-activated MФ subpopulations. However, surprisingly, TNF-α levels were differently affected in the two MФs. While ATRA increased NOD1-induced TNFα secretion in M-MФs, the secretion of this cytokine was significantly attenuated in GM-MФs by ATRA pretreatment. Interestingly, opposite effect was detected for IL-8 production, as ATRA highly down-regulated the secretion in M-MФs, while significantly enhanced secretion was observed in the supernatant of ATRA-treated NOD1-activated GM-MФs. Surprisingly, ATRA also appeared to polarize release of the anti-inflammatory IL-10 and IFNβ production; (**Supplementary Figure 1C**) in an opposite direction to IL-6 and TNF-α, and its levels were decreased by ATRA in GM-MФs, while it was increased in M-MФs, similarly to IL-8. Altogether, these results show that ATRA highly modulates NOD1-induced cytokine secretion in human MФs, and this modulation is cytokine- and cell type-dependent.

### NOD2 induces cytokine secretion by different human MФ subpopulations with similar tendency as NOD1

**Figure 2:**
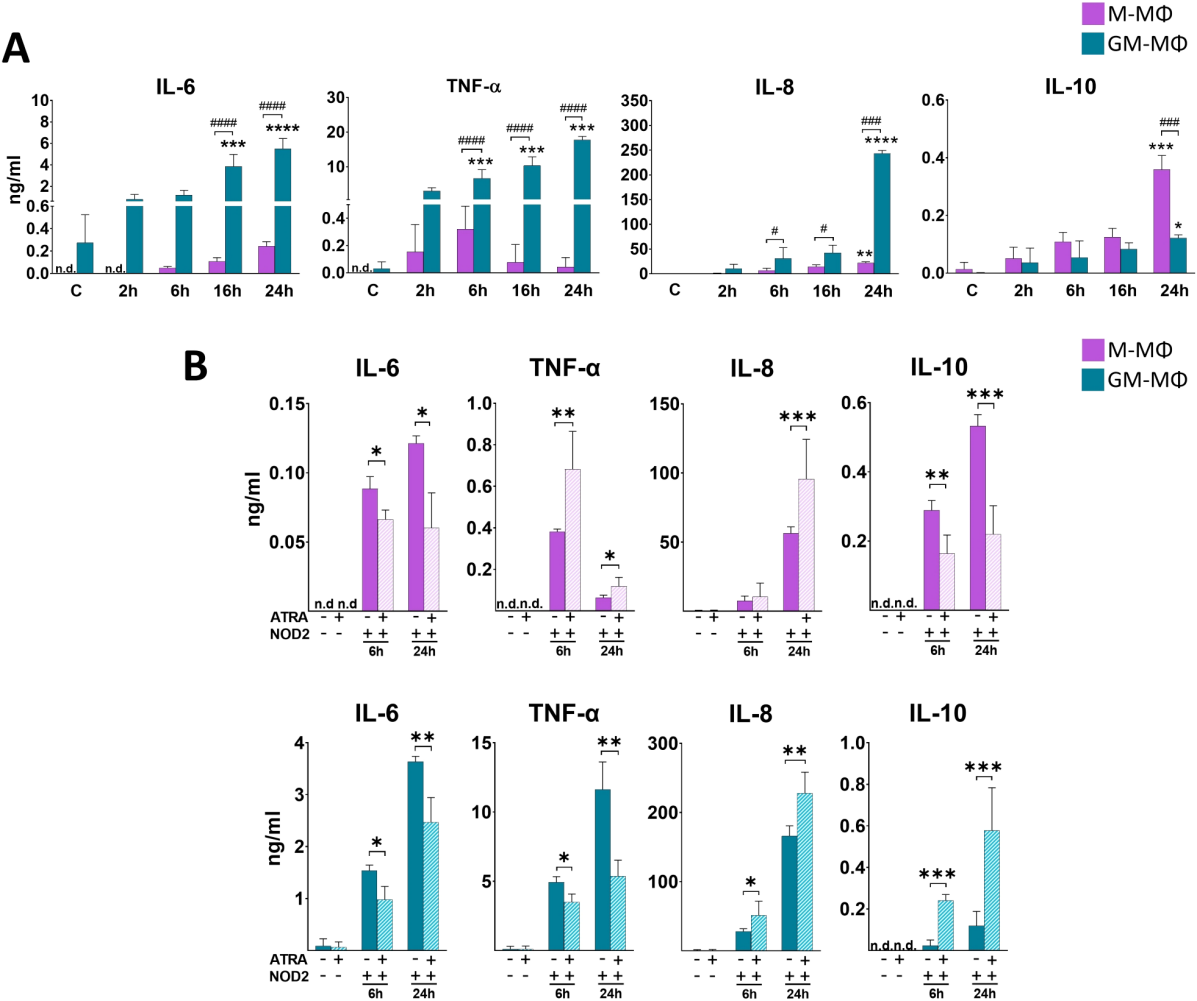
Secretion of IL-6, TNF-α, IL-8 and IL-10 following NOD2 activation. M-MФ and GM-MФ were treated with L-18 MDP (NOD2 agonist, 100 ng/ml) for the indicated time points. Control cells were treated with the same amount of vehicle as the activated cells. Cytokine secretion was measured from the supernatant using ELISA. **(A)** Time kinetics of cytokine secretion. **(B)** Cells were pretreated with ATRA (1µM) for 4 hours and then stimulated with L-18 MDP for 6 and 24 hours. Results were obtained from at least four healthy donors. All results are shown as means ± SD. (^*^p < 0.05, ^**^p < 0.01, ^***^p < 0.001, ^****^p < 0.0001, ^#^p < 0.05, ^###^p < 0.001, ^####^p < 0.0001; n.d., not detected).

While NOD1 specifically recognizes peptidoglycan components of Gram (–) bacteria, NOD2 - a very close family member of NOD1 - is able to recognize peptidoglycan pattern from both Gram (–) and Gram (+) bacteria. Hence, next we aimed to determine the cytokine secretion by the two MФ subpopulations following NOD2 activation. Cells were treated with NOD2 agonist L18-MDP, and supernatant was collected for cytokine measurement (**Figure 2A**). While we measured higher level of NOD2-induced IL-6 and TNF-α in GM-MФs compared to the M-MФs, NOD2 treatment resulted in a gradually increasing TNF-α secretion during the 24 h treatment in GM-MФs, in contrast to the 12 h peak of TNF-α by the NOD1-induced cells. Similar to NOD1 activation, NOD2 treatment resulted in robust IL-8 secretion in GM-MФs, while it was released at a moderate level by M-MФs. NOD2 activation resulted in higher IL-10 secretion in M- MФs compared to GM- MФs, similar to that observed with NOD1 activation. IFN-β release was gradually increasing and comparable between the MФ populations (**Supplementary Figure 1B**).

### In M-MФs, ATRA differently modifies NOD2-induced IL-8 secretion compared to induction by NOD1

Then, we aimed to see the potential modulatory effect of ATRA on NOD2-induced cytokines production. Similar to NOD1, in the presence of ATRA we detected IL-6 decrease both in M-MФs and GM-MФs following NOD2 activation (**Figure 2B**). Similar to NOD1, opposite effects were observed for TNF-α and IL-10 secretion between the two cell types; while ATRA enhanced TNF-α and decreased IL-10 secretion in M-MФs, ATRA decreased TNF-α and enhanced IL-10 production in GM-MFs. Interestingly, however, IL-8 chemokine secretion was significantly upregulated by ATRA in both M- and GM-MФs, in contrast to the NOD1-induced IL-8 secretion in M-MФs, where ATRA downregulated the cytokine secretion. Again, similar tendency was measured for IL-10 secretion (and IFNβ production; (**Supplementary Figure 1D**)); the anti-inflammatory cytokine was decreased by ATRA in M-MФs, while it was increased in GM-MФs.

### ATRA differently modifies NOD1 ligand-induced IL-1β and IL-18 secretion in the human MФ subpopulations

**Figure 3:**
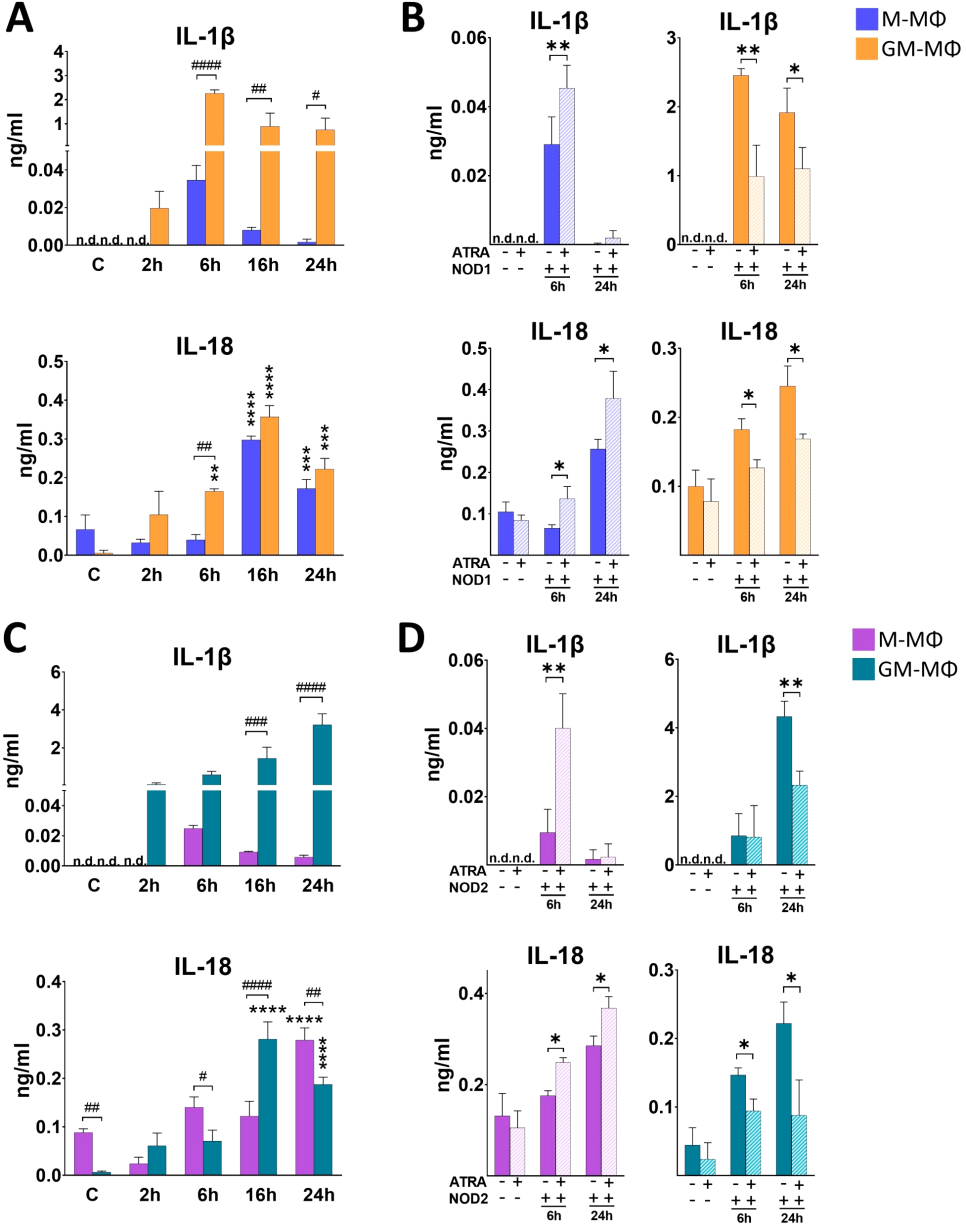
Secretion of IL-1β and IL-18 following NOD1 or NOD2 activation. M-MФ and GM-MФ were treated with **(A, B)** C14-Tri-LAN-Gly (NOD1 agonist, 500 ng/ml) or **(C, D)** L-18 MDP (NOD2 agonist, 100 ng/ml) for the indicated time points. Control cells were treated with the same amount of vehicle as the activated cells. Cytokine secretion was measured from the supernatant using ELISA. **(A, C)**) Time-kinetics of cytokine secretion. **(B, D)** Cells were pretreated with ATRA (1µM) for 4 hours and then stimulated with NOD1 or NOD2 agonist for 6 and 24 hours. Results were obtained from at least four healthy donors. All results are shown as means ± SD. (^*^p < 0.05, ^**^p < 0.01, ^***^p < 0.001, ^****^p < 0.0001, ^#^p < 0.05, ^##^p < 0.01, ^###^p < 0.001, ^####^p < 0.0001; n.d., not detected).

In contrast to the other cytokines, the maturation of pro-IL-1β and pro-IL-18 pro-inflammatory cytokines to their active form requires proteolytic cleavage by caspase enzymes. Interestingly, cell activation with NOD1 agonist treatment has been implicated in IL-1β/IL-18 secretion (12). To determine if activation of the monocyte-derived MФs with NOD1 agonist induce their secretion, supernatant was collected for ELISA measurements at different time points following NOD1 agonist treatment. We observed a rapid induction with an early peak (6 h) in IL-1β secretion in GM-MФs, while IL-1β secretion by M-MФs was hardly detectable (**Figure 3A**). In the case of IL-18, the secretion peaked at 16 h in both MФs, and the secretion level of IL-18 was comparable in the two MФ populations (**Figure 3A**). However, while in M-MФs both IL-1β and IL-18 secretion showed increasing tendency in the presence of ATRA, we observed an opposite effect in GM-MФs, as the secretion of both IL-1β and IL-18 was significantly attenuated by ATRA pretreatment (**Figure 3B**).

### NOD2-induced IL-1β and IL-18 secretion are also differently modified by ATRA in the human MФ subpopulations

Then, we aimed to see whether NOD2-activation induces IL-1β and IL-18 secretion, and if the amount and dynamics of these cytokine secretions are comparable with what we observed in the case of NOD1 activation. We found that NOD2 agonist treatment resulted in a gradual increase of both IL-1β and IL-18 in GM-MФs (**Figure 3C**). Similar to NOD1, however, IL-1β secretion by M-MФs was hardly detectable. Also, similar to NOD1, while ATRA enhanced NOD2-induced IL-1b and IL-18 secretion in M-MФs, the production of both cytokines was decreased in GM-MФs in the presence of ATRA (**Figure 3D**).

## Discussion

Macrophages are remarkably plastic immune cells, whose functional characteristics are highly determined by their origin, and shaped by the tissue microenvironment of their location, as well as the activating stimuli that trigger their effector functions (25, 26). *In vitro*, differentiation of macrophages from human primary monocytes using GM-CSF or M-CSF are widely used methods to study macrophage polarization and functions, as the generated GM-MФs or M-MФs possess inflammation inducing or inflammation resolving macrophage characteristics, respectively (27, 28). Here we show that the activation of these primary monocyte-derived MФ subpopulations via NOD1 and NOD2 cytosolic receptors results in characteristic profile of secreted cytokines that are typically released during macrophage inflammatory responses, and contribute to pro-inflammatory or pro-resolving/tissue regenerating mechanisms. We detected significantly higher release of the inflammatory IL-6, TNF-α, IL-8 and IL-1β cytokines from the pro-inflammatory GM-MФs compared to the pro-resolving M-MФs. However, the secretion of IL-18 pro-inflammatory cytokine was comparable between the two cell types. Furthermore, although the level of anti-inflammatory IL-10 was more pronounced in the M-MФs compared to the GM-MФs, the level of IL-10 secretion was also in a comparable range between the two subtypes. These results highlight that depending on the type of MФs affected, NOD1/NOD2-mediated cytokine secretion may drive potentially different induction and polarization of cells which is also likely to shape their interactions with cells of adaptive immunity.

NOD1 and NOD2 are the first described members of the cytosolic pattern recognition NOD-like receptor family. Upon recognition of respective ligands by NOD1 or NOD2 induces recruitment of RIPK2 which then mediates the activation of NF-kB and MAPK (ERK, p38, JNK) signaling pathways and drives various cellular processes including cytokine/chemokine secretion (29). RIPK2 activity may also induce the nuclear translocation of IRF7 transcription factor to promote type I IFN release (29, 30) which is an efficient upstream signaling cytokine of IL-10. Furthermore, RIPK2 may also recruit and directly bind caspase-1, which is one of the major regulatory enzymes of IL-1β and IL-18 secretion (10). Besides, NOD1/NOD2 may interact with other, inflammasome forming Nod-like receptors to induce IL-1β and IL-18 secretion (12). However, our comparative analysis also reveals substantial differences in the amount of the secreted typical cytokines between the MФ subtypes, indicating that activation of NOD1/NOD2 results in a subpopulation characteristic pattern of cytokine secretion.

**Figure 4:**
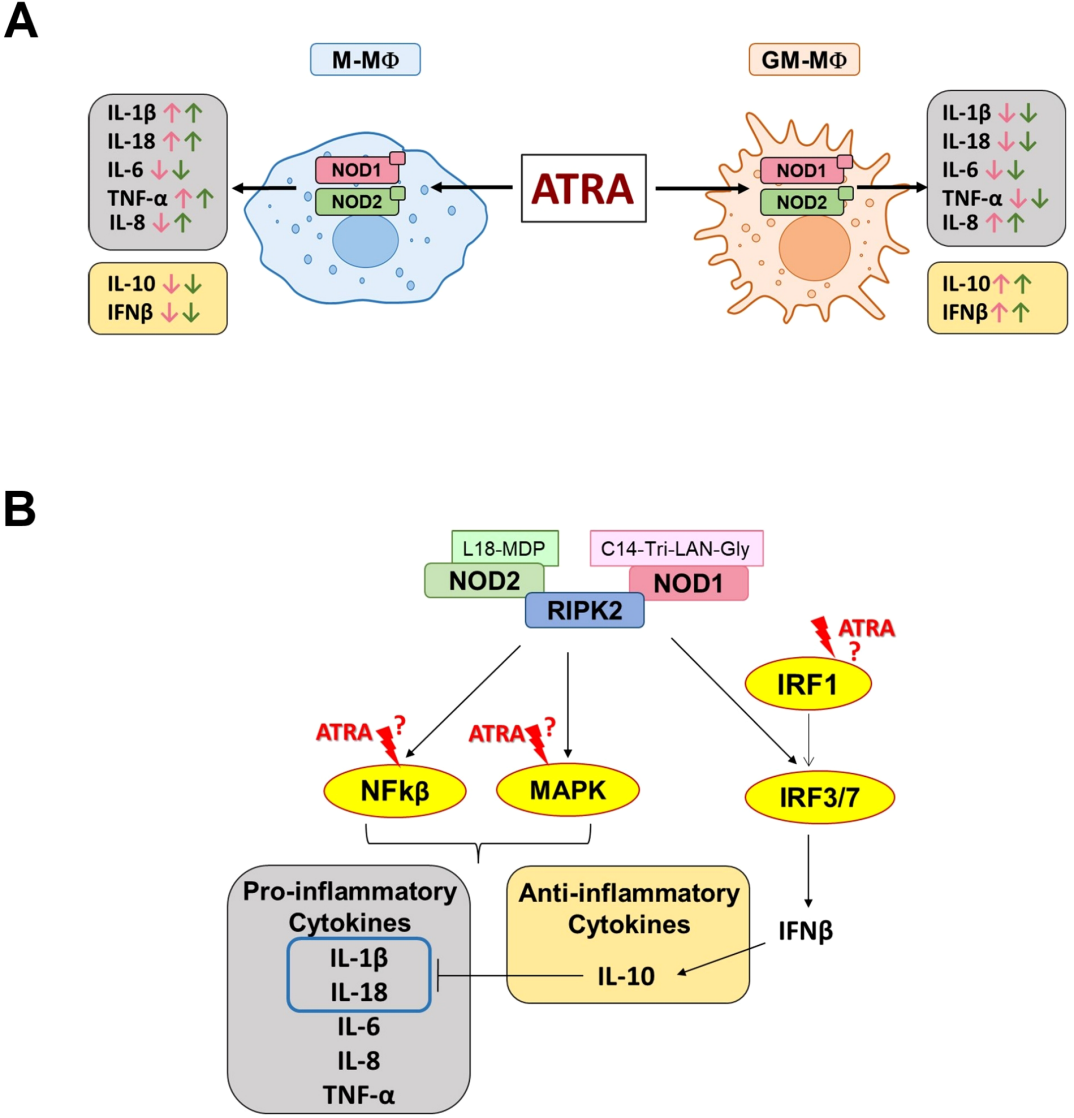
Schematic representation of ATRA-mediated effects on NOD1/NOD2-induced cytokine secretion. **(A)** Summary of the modulatory effect of ATRA on the NOD1/NOD2-induced cytokine secretion in the different subpolulations of human macrophages. Pink and green arrows indicate NOD1- and NOD2-induced cytokine changes, respectively. M-MФ (M-CSF-differentiated MФ), GM-MФ (GM-CSF-differentiated MФ). **(B)**. Potential intervention points of ATRA on the NOD1/NOD2-induced signaling pathways leading to cytokine secretion. Following activation, NOD1/NOD2 recruits RIPK2 enzyme which may trigger the activation of various signal transduction pathways leading to the secretion of cytokines (29, 30).

Importantly, these results are in line with our previous findings on LPS-activated MФ subtypes (31, 32). However, while LPS is a harsh activator that is detected by TLR4 and drives high level of IL-10 secretion from M-MФs and high level of pro-inflammatory cytokine from GM-MФs, NOD1/NOD2 induce milder effects and results in a less pronounced secretion of the corresponding cytokines. Importantly, however, we show now that the most highly induced cytokine by NOD1/NOD2 activation is the pro-inflammatory interleukin-8 (IL-8). Surprisingly, NOD1/NOD2-induced IL-8 levels are similar to what we reported following LPS stimulation from these types of cells (26, 27). IL-8 is a crucial regulator of angiogenesis and a potent neutrophil chemotactic factor, hence plays an important role in physiological and pathological conditions (33). It was shown that at low concentrations, IL-8 gradient attracts neutrophils to the inflammatory foci, while at high, receptor-saturating concentration, it triggers neutrophils to release granule proteins and chromatin to form neutrophil extracellular traps (NETs) (33, 34). Hence, we speculate that while both MФ populations possess high capability of neutrophil attraction, NOD1/NOD2-activated GM-MФs may even have good potency to induce NET formation. RA, the most common, physiologically active metabolite of vitamin A, appears as an important tissue-derived signal that contributes to MФ polarization. However, the presence and distribution of RA shows high variability between the organs and even within a tissue, providing different microenvironments to the MФs. While NOD1 and NOD2 are one of the main cytosolic pathogen recognition receptors of MФs, the potential modulatory effect of ATRA on NOD1/NOD2-mediated responses by the different MФ subpopulations has not been addressed before. Here, we show that ATRA significantly modifies cytokine and chemokine release induced by NOD1 and NOD2 activation, and importantly, ATRA treatment results in various effects in the cytokine secretion (**Figure 4A**). In M-MФs, we observed that the NOD1-induced pro-inflammatory TNF-α, IL-1β and IL-18 were upregulated, while IL-6 and IL-8 were down-regulated by ATRA. Furthermore, the NOD1-induced anti-inflammatory IL-10 was also decreased by ATRA. In GM-MФs, however, under the same conditions, we observed completely the opposite effect of ATRA on cytokine secretion, with the exception of IL-6. Indeed, IL-6 was the only cytokine that, in each condition, was affected the same way by ATRA, as it was down-regulated in both MФ subtypes following both NOD1 and NOD2 activation. Importantly, NOD2-induced cytokine secretions were affected with the same tendency by ATRA as in the case of NOD1 activation, except for IL-8, as the secretion of this cytokine was enhanced both in M-MФs and GM-MФs. Cytokines released by MФs may act in an autocrine or paracrine manner to regulate the secretion of other cytokines. Like IFNβ is an upstream regulator of IL-10 (20), and both IFNβ and IL-10 are negative regulators of caspase-1-mediated IL-1β secretion (35, 36), which may explain the negative correlation between IFNβ/IL-10 and IL-1β/IL-18 secretion by the MФs in the presence of ATRA in our results. Of note, it was reported that ATRA induces the expression of IRF1 transcription factor in human myeloid cell lines and MФs (37). On the other hand, IRF1 was identified as a transcription regulator of Interferon-regulated genes (ISG) by facilitating chromatin accessibility for IRF3 binding which is a major driver of IFNβ expression (38). Nevertheless, ATRA-induced engagement of IRF1 in IFNβ secretion requires further comparative analysis in these MФ subpopulations.

ATRA may be recognized by various nuclear receptors (mainly by RAR, but also by PPAR or ROR) that may directly regulate gene expression in the nucleus (39). However, non-genomic effects of nuclear receptors have also been described, as in the cytosol, they can directly interact and modulate proteins of various signal transduction pathways (such as, AKT, NF-κB and ERK) (40). Importantly, many of these pathways overlap with those that are also affected by NOD1/NOD2 activation, rising the potential possibility of potential cross -talk mechanisms. Previous studies have reported context-dependent effect of ATRA on the expression of Toll-like receptors (TLRs) and RIG-like helicases (RLHs). Like ATRA downregulated the expression of TLR2 in monocytes (41) and TLR4 in the inflamed lung (6), while it upregulated TLR5 in THP-1 cells (42) and TLR4 expression in CaCo2 cells (43). ATRA alone upregulated TLR3, TLR4 and RIG-I intestinal epithelial cells, however it suppressed TLR3, TLR7 and RIG-I expression following viral infections (44, 45). Despite these observations, the mechanism of ATRA action has not been revealed. Regarding NLRs, the effect of ATRA has only been studied on the NLRP3 inflammasome activation. It has been reported that ATRA supplementation reduces the expression of NLRP3 inflammasome and caspase-1 in the brain tissue of a chronic alcoholic rat model (46). While, in our previous studies using *in silico* method, we found putative RAR-response elements in the promoter of NLRP3 and we detected increased NLRP3 protein level following ATRA treatment alone in human M-MФs. Furthermore, we found that ATRA shifts M-MФs metabolism toward glycolysis (19), which can highly effect MФs polarization. In this current study, we didn’t detect changes in the mRNA expression of NOD1/NOD2 (data not included) nor in the cytokine secretion following ATRA treatment alone, which suggests that ATRA acts as a modulator of NOD1/NOD2-induced cytokine production via downstream actions (and/or overall metabolic status of the cell) rather than inducing cytokine production alone.

It has been reported that ATRA treatment modulates several major intracellular signaling pathways, including MAPK signaling (such as ERK, JNK and p38). In human scleral fibroblasts, ATRA decreased ERK1/2 activation, while it increased JNK activation (47), however, in human MФs, ATRA enhanced the phosphorylation of ERK and attenuated that of p38 (19). The ATRA effect on NF-κB is also controversial, as it exhibits an inhibitory effect in THP-1, alveolar MФs and epithelial cells (6, 48), while in human keratinocytes, it induces the activation of both the NF-κB and p38 pathways (49). ATRA can also inhibit the PI3K/AKT signaling in human fibroblasts (50), and induces IRF-1 expression and its nuclear localization in human MФs (37, 51). Importantly, many of these pathways overlap with those that are also affected by NOD1/NOD2 activation, rising the possibility of cross-talk mechanisms (**Figure 4B**).

## Conclusions

In summary, our results show that NOD1/NOD2-induced cytokine secretion by macrophages (MФ) is cell type dependent and it is highly modified by the nuclear receptor agonist ATRA. The implications of these observations are far reaching. NOD1 and NOD2, as sensors of microorganisms and cellular stress, have been identified as potential therapeutic targets in a range of autoimmune and autoinflammatory disorders (52) and in diabetes, asthma, atherosclerosis, various forms of CNS diseases and even cancer (29). In line with this, while it is well established that vitamin A and retinoic acid play important homeostatic and regulatory roles in multiple tissues, based on our data, MФs activated by NOD1/NOD2-dependent or independent stimuli are likely to be profoundly modulated by ATRA. Major immune organs also require a steady dietary intake of vitamin A to maintain their functions and vitamin A deficiency has been associated with dysbiosis, frequent infectious diseases and reduced immune response to vaccines, although the underlying mechanisms have remained unclear to date. Our data shed light on this issue, by showing the potent and diverse modulatory role of ATRA on NOD1/NOD2-induced cytokine secretion. Based on previous reports, we suggest that this modulation is mediated, in part, via interrelated signaling pathways and altered metabolic status of the cell. Hence, we speculate that ATRA may function as a general regulator of cytokine secretion induced by various PRRs. While understanding the precise mechanism of ATRA action on NOD1/NOD2-induced mechanisms in different tissues and MФ populations requires further studies, our results highlight the therapeutic relevance of these pathways in a number of inflammatory conditions, which represent an increasing burden to society.

## Data Availability

The original contributions presented in the study are included in the article/**Supplementary Material**. Further inquiries can be directed to the corresponding author.

## Supplementary material

Supplementary Figure 1 Secretion of IFNβ following NOD1 and NOD2 activation. M-MФ and GM-MФ were treated with C14-Tri-LAN-Gly (NOD1 agonist, 500 ng/ml) and L-18 MDP (NOD2 agonist, 100 ng/ml) for the indicated time points. Control cells were treated with the same amount of vehicle as the activated cells. Cytokine secretion was measured from the supernatant using ELISA. Time kinetics of IFNβ secretion following **(A)** NOD1 activation and **(B)** NOD2 activation. Cells were pretreated with ATRA (1µM) for 4 hours and then stimulated with **(C)** C14-Tri-LAN-Gly or **(D)** L-18 MDP for 6 and 24 hours. Results were obtained from at least four healthy donors. All results are shown as means ± SD. (* p < 0.05, ** p < 0.01, *** p < 0.001, **** p < 0.0001, # p < 0.05, ## p < 0.01, ### p < 0.001, #### p < 0.0001; n.d. – not detected).
