# Supplementary figures and images for: Retinoic acid differently modulates NOD1/NOD2-mediated inflammatory responses in human macrophage subsets
Source: Front Immunol. 2025 Jul 1;16:1609763. doi: 10.3389/fimmu.2025.1609763 (PMC12259451; doi:10.3389/fimmu.2025.1609763)

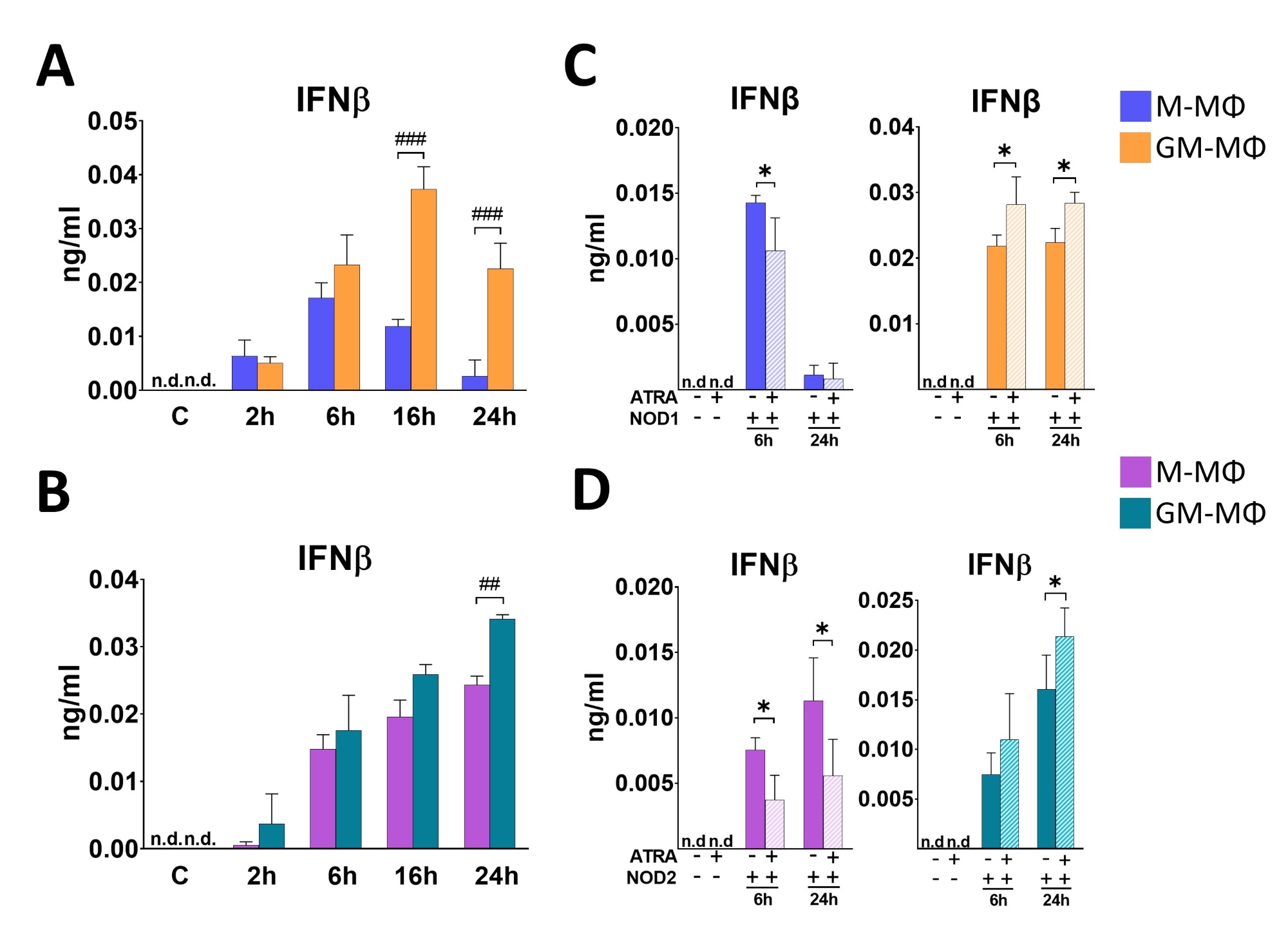

Supplement: Supplementary Figure 1 — Secretion of IFNβ following NOD1 and NOD2 activation. M-MФ and GM-MФ were treated with C14-Tri-LAN-Gly (NOD1 agonist, 500 ng/ml) and L-18 MDP (NOD2 agonist, 100 ng/ml) for the indicated time points. Control cells were treated with the same amount of vehicle as the activated cells. Cytokine secretion was measured from the supernatant using ELISA. Time kinetics of IFNβ secretion following (A) NOD1 activation and (B) NOD2 activation. Cells were pretreated with ATRA (1µM) for 4 hours and then stimulated with (C) C14-Tri-LAN-Gly or (D) L-18 MDP for 6 and 24 hours. Results were obtained from at least four healthy donors. All results are shown as means ± SD. (* p < 0.05, ** p < 0.01, *** p < 0.001, **** p < 0.0001, # p < 0.05, ## p < 0.01, ### p < 0.001, #### p < 0.0001; n.d. – not detected). [file Image1.jpeg]
